# Characteristic Analysis and Structural Design of Hollow-Core Photonic Crystal Fibers with Band Gap Cladding Structures

**DOI:** 10.3390/s21010284

**Published:** 2021-01-04

**Authors:** Bowei Wan, Lianqing Zhu, Xin Ma, Tianshu Li, Jian Zhang

**Affiliations:** 1 Beijing Laboratory of Optical Fiber Sensing and System, Beijing Information Science and Technology University, Beijing 100016, China; 06356@buaa.edu.cn (B.W.); zhangjian0112@bistu.edu.cn (J.Z.); 2 School of Instrument Science and Opto-Electronics Engineering, Hefei University of Technology, Hefei 230009, China; litianshu@mail.hfut.edu.cn; 3 School of Instrument Science and Optoelectronic Engineering, BeiHang University, Beijing 100191, China; maxin@buaa.edu.cn

**Keywords:** hollow-core photonic crystal fiber, photonic band gap effect, finite element method, COMSOL, characteristic analysis, structural design

## Abstract

Due to their flexible structure and excellent optical characteristics hollow-core photonic crystal fibers (HC-PCFs) are used in many fields, such as active optical devices, communications, and optical fiber sensing. In this paper, to analyze the characteristics of HC-PCFs, we carried out finite element analysis and analyzed the design for the band gap cladding structure of HC-PCFs. First, the characteristics of HC19-1550 and HC-1550-02 in the C-band were simulated. Subsequently, the structural optimization of the seven-cell HC-1550-02 and variations in characteristics of the optimized HC-1550-02 in the wavelength range 1250–1850 nm were investigated. The simulation results revealed that the optimal number of cladding layers is eight, the optimal core radius is 1.8 times the spacing of adjacent air holes, and the optimal-relative thickness of the core quartz-ring is 2.0. In addition, the low confinement loss bandwidth of the optimized structure is 225 nm. Under the transmission bandwidth of the optimized structure, the core optical power is above 98%, the confinement loss is below 9.0 × 10^−3^ dB/m, the variation range of the effective mode field area does not exceed 10 μm^2^, and the relative sensitivity is above 0.9570. The designed sensor exhibits an ultra-high relative sensitivity and almost zero confinement loss, making it highly suitable for high-sensitivity gas or liquid sensing.

## 1. Introduction

**Figure 1:**
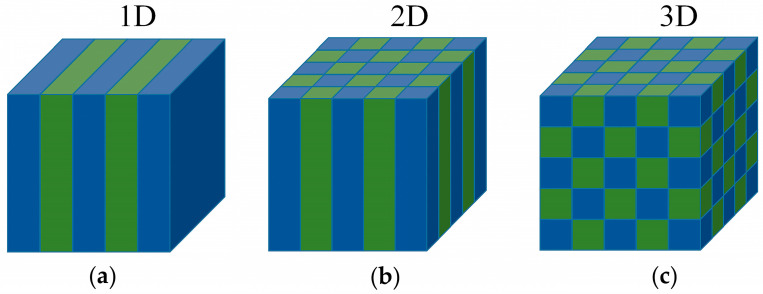
(**a**) One-dimensional (1D), (**b**) two-dimensional (2D) and (**c**) three-dimensional (3D) photonic crystal structures.

Photonic crystals, first proposed by Yablonovitch in 1987, are a medium with a periodic distribution of the refractive index (RI) in space [1]. According to the periodic arrangement of the constituent medium, photonic crystals can be classified into three structure types: one-dimensional (1D) (e.g., fiber grating), two-dimensional (2D), and three-dimensional (3D) [2], as shown in Figure 1.

Photonic crystal fibers (PCFs) are composed of 2D photonic crystals with a lattice constant of the order of the optical wavelength, and are also known as holey fibers or microstructure fibers (MFs). In 1996, Knight et al. [3] prepared PCFs in the laboratory for the first time, which promoted the development of PCFs in the signal transmission field. The biggest difference between PCFs and other MFs is that there is a defect in the core, which destroys the periodic arrangement of the cladding structure; thus, the different RIs of defective materials affect the transmission characteristics of a PCF.

According to the optical transmission principle of PCFs, they can generally be divided into two types: total internal reflection PCFs (TIR-PCFs) and photonic band gap PCFs (PBG-PCFs). For PBG-PCFs, the optical transmission principle is unlike the TIR optical transmission principle of a traditional optical fiber. It is guided by the PBG effect; that is, the cladding structure has a band gap effect for photons at a certain frequency range, and the beam can only be transmitted in the core [4]. PBG-PCFs have strict requirements in terms of the shape and arrangement of cladding air holes, so that the RI of the optical fiber cladding cross-section has a periodic distribution. Due to the PBG effect, most of the light is bound to achieve low confinement loss transmission in a large air hole with low RI.

Knight et al. [5] first prepared PBG-PCFs with a honeycomb cladding structure in 1998. Soon after, Roberts et al. [6] reduced the lowest confinement loss of PBG-PCFs at 1565 nm to 1.2 dB/km, with 98.3% of its photon energy restricted to the large air hole, 1% to the cladding holes, and only 0.7% to the quartz. In addition, Chen [7] carried out a simulation analysis of the optical characteristics of all-solid-state refraction-guiding PCFs, including mode characteristics, loss characteristics, and dispersion characteristics. In 2010, Buczynski et al. [8] used lead–bismuth–gallium silicon glass to make PCFs with a working wavelength of 700–2500 nm, and successfully obtained a supercontinuum from near-infrared to mid-infrared. At the same time, Vieweg et al. [9] filled PCFs with a highly nonlinear liquid and obtained a continuous spectrum with a bandwidth of more than 600 nm. Research has been carried out on the achievement of novel plasma biosensors by selectively incorporating metals and 2D materials into the cladding air holes [10,11]. In 2019, Fan et al. [12] proposed an analysis and simulation of a PCF optical sensor based on nano-gold ring film coverage. When the RI was from 1.40 to 1.43, the sensor had a high linear sensitivity of 3978 nm/RIU. Li et al. [13] recently obtained an H-type PCF surface plasmon resonance sensor that was coated with an Ag-graphene layer. In the linear RI sensing area of 1.33–1.36, this sensor had an average wavelength sensitivity of 2770 nm/RIU and a resolution of 3.61 × 10^−5^ RIU.

There have been many studies on the characteristic analysis and structural design of hollow-core PCFs (HC-PCFs) in recent years. Gao et al. [14] proposed an ultra-wide-bandwidth negative-curvature HC-PCF, which achieved the lowest confinement loss of 2 dB/km at 1512 nm and a communication bandwidth of up to 335 nm with a confinement loss of below 16 dB/km. At the same time, Jia et al. [15] proposed a PCF with a hollow-ring structure that could support 38 kinds of orbital angular momentum mode transmission. With the increase of the wavelength, the confinement loss under this structure ranged from 10^−10^ to 10^−9^ dB/m. Soon after, Li et al. [16] proposed a novel plasmonic nodeless HC-PCF, the polarization filtering characteristics of which were investigated in detail. Gao et al. [17] recently proposed an ultra-low-loss HC-PCF with an isolated anti-resonance layer, which achieved a confinement loss of below 3.5 dB/km at 1550 nm by using a 13 μm core diameter and an 8 μm effective mode field diameter. In addition, the bending loss and effective mode field area of HC-PCF were studied by Frosz et al. [18] and Ma et al. [19], respectively. HC-PCFs have also been widely studied in the field of trace gas or liquid sensing [20,21]. In recent years, an HC-PCF gas sensing system based on spectroscopy technology has been able to achieve high precision gas sensing at the ppb level [22,23]. Lin et al. [24] proposed distributed and quasi-distributed gas sensing based on a seven-cell HC-PCF. Photothermal technology was applied to distributed gas detection to achieve a higher performance optical fiber distributed system in this scheme. At the same time, Tan et al. [25] used a highly reflective fiber end mirror at one end of an HC-PCF to achieve multiple superposition of the effective optical path length, which was conducive to improving the sensitivity of the gas sensing system. Quintero et al. [26] recently used a 74-cm-long HC-PCF as the gas chamber to achieve an all-fiber CO_2_ sensor.

The existing literature indicates that PCFs have significant application value in many fields such as gas sensors [27,28] and as ultra-wideband solar absorbers [29,30]. Due to their flexible structure and excellent optical characteristics. However, the relationship between the structure and performance of PCFs is not clear, restricting the application of new generation PCFs. Due to the special structure of HC-PCFs with multiple air holes, it has higher confinement loss than an ordinary single-mode fiber. Therefore, the structure of HC-PCFs should be designed and optimized to achieve ultra-low confinement loss and long transmission bandwidth in the communication band [31]. In this study, finite element modeling and simulation were carried out to analyze the characteristics of HC-PCFs. First, the characteristics of HC19-1550 and HC-1550-02 were analyzed. Then, considering the advantages associated with the simple production process, low cost, and wide application of seven-cell HC-1550-02, we focused on the optimization design of its structure. The core radius and the core quartz-ring thickness were determined, and the optimal structure was obtained. Finally, the optimized HC-1550-02 was used to analyze the variation trends of core optical power, confinement loss, and effective mode field area in the transmission band.

## 2. Characteristic Analysis of HC-PCF

### 2.1. Characteristic Analysis of Normalized Frequency and Infinite Single-Mode Transmission

The normalized frequency *V* determines the mode numbers of optical fiber transmission. For a traditional step fiber, single-mode transmission is defined as follows:

(1) $$V=\frac{2\pi \rho }{\lambda }\sqrt{n_{\mathrm{co}}^{2}-n_{\mathrm{cl}}^{2}}$$

where *n*_co_ and *n*_cl_ are the RI of the core and cladding, respectively, *ρ* is the core radius, and *λ* is the wavelength of the optical transmission.

For traditional optical fiber, the cladding’s RI is almost independent of the wavelength. When the wavelength decreases, the normalized frequency *V* increases, where its normalized cut-off frequency is 2.405. Unlike traditional optical fibers, the core of PCF is composed of a defect that periodically destroys the cladding structure [32]. Therefore, for PCF, the normalized frequency *V* is defined as follows:

(2) $$V=\frac{2\pi \Lambda }{\lambda }\sqrt{n_{eff}^{2}-n_{\mathrm{FSM}}^{2}}$$

where *n_eff_* and *n*_FSM_ represent the effective RI of the core fundamental mode and the cladding fundamental space-filling mode (FSM), respectively, and *Λ* is the spacing of adjacent air holes.

It can be seen from Equation (2) that *V* is not only related to the wavelength, but is also related to *Λ* and the diameter *d* of the cladding air hole. Birks et al. [33] described the relationship between *V* and *Λ*/*λ*. Briefly, when *Λ*/*λ* tends to infinity, the wavelength tends to 0, while *V* tends to be constant. Mortensen et al. [34] discussed the relationships between *V* and *Λ*/*λ*, *V* and *λ*/*Λ*, and *V* and *d*/*Λ* in HC-PCF. These studies showed that when *V* < π or *d*/*Λ* < 0.406, PCF could achieve infinite single-mode transmission without the cut-off wavelength.

### 2.2. Characteristic Analysis of Effective Mode Field Area and Core Optical Power

In HC-PCFs, the photon energy is not completely concentrated in the central air hole for transmission, and some photon energy is transmitted to the cladding. Saitoh et al. [35] proposed the concept of the effective mode field area *A_ef__f_*, and its calculation formula is presented as follows:

(3) $$A_{eff}=\frac{{\left[\iint \left|E{\left(x,y\right)}^{2}\right|dxdy\right]}^{2}}{\iint \left|E{\left(x,y\right)}^{4}\right|dxdy}$$

where *E*(*x*, *y*) is the electric field intensity distribution of the optical fiber fundamental mode. In the finite element simulation, the area integral can be used to integrate *E*(*x*, *y*). In addition, the order of integration corresponds to the numerator and denominator in Equation (3), and the ratio of the final derived data is *A_eff_*.

When HC-PCFs are applied in a gas or liquid sensing system, the sensing sensitivity of the system is proportional to the overlap ratio of the physical quantity to be measured and the photon field; that is, the higher the ratio of the optical power in the sensing areas of all air holes to the total optical power in the cladding end-face of the HC-PCF, the higher the sensing sensitivity. This ratio is also known as the optical power distribution function, *f*, and it can be calculated by [36]:

(4) $$f=\int_{\mathrm{holes}}\left(E_{x}H_{y}-E_{y}H_{x}\right)dxdy/\int_{\mathrm{total}}\left(E_{x}H_{y}-E_{y}H_{x}\right)dxdy$$

where *E_x_*, *E_y_*, *H_x_* and *H_y_* are the *x* and *y* components of the electric and magnetic fields in the cross-section of the optical fiber mode, respectively. In finite element simulation, the global computing module is used to solve Maxwell’s equations. First, the surface integral of the sensing areas of all air holes and the cladding end-face of the HC-PCF are defined. Then, the time-averaged power flow *z*-component of the electric field in the corresponding integral area is calculated. The final ratio is *f*.

### 2.3. Characteristic Analysis of Confinement Loss

There are various optical transmission losses in PCF, such as the surface mode coupling loss, which is a defect mode that usually appears in the cross-section of the periodic crystal structure. In PBG-PCFs, the surface mode appears on the cross-sections of the core and the cladding. Through this surface mode, the photon energy of the core transmission mode can be coupled into the cladding, which leads to the coupling loss of the optical fiber surface mode [37]. A reasonable design of the core structure can avoid the appearance of the surface mode [38]. In addition to the surface mode coupling loss, the confinement loss *α*_loss_ is also an indispensable factor in PCFs. This is due to the limitation of the number of air holes and the number of layers in the cladding, which cannot restrict all of the light in the core, resulting in a loss caused by partial light leakage to the cladding. The calculation formula for the confinement loss is presented as follows [39]:

(5) αloss=8.686×2π×10−9λ[nm]Im(neff)[dB/m]

It can be seen from Equation (5) that *n_eff_* must have an imaginary part when calculating the confinement loss. Different values for the diameter, spacing and arrangement of air holes in the cladding, or increasing layer numbers of air holes in the cladding, can significantly reduce or even allow one to ignore the confinement loss factor.

## 3. Finite Element Modeling and Simulation of HC-PCF

Considering that HC-PCFs are used in trace gas or liquid detection where the detected gas or liquid may be toxic or explosive, the trace gas or liquid detection system must be highly sensitive within the safety detection range. Therefore, it is necessary to establish a theoretical model for HC-PCFs, analyze their transmission characteristics, and determine their optimal structure.

### 3.1. Finite Element Modeling

The finite element method (FEM) is the most commonly used method to solve and analyze electromagnetic field problems. FEM has strong flexibility and stability, and shows great advantages in HC-PCF simulation of complex structures. It is the most commonly used and accurate calculation method available at present.

As an optical fiber belongs to an open waveguide, its mode field is distributed in an infinite space. Restricted by computing power and practical conditions, the numerical calculation can only be carried out in a limited area. Therefore, some appropriate boundary conditions (BCs) are normally used to simulate the truncated infinite space. A suitable BC not only improves the calculational accuracy, but also improves the calculational efficiency and saves computing resources. Therefore, the selection of BCs is very important for efficient numerical calculation. Perfectly matched layer (PML) is often used as an ideal BC in the FEM analysis of HC-PCFs. PML was a concept proposed by Berenger [40] in 1994. The basic idea is to set up a dielectric layer at the truncated boundary that is perfectly matched with the wave impedance of the adjacent dielectric so that the incident wave enters the PML without reflection. A PML is generally composed of conductive and magnetic media, which can absorb incident electromagnetic waves of any incident angle, frequency, and polarization state; thus, it is equivalent to an almost ideal absorber or radiator.

### 3.2. Characteristic Analysis of HC-PCF Mode

**Figure 2:**
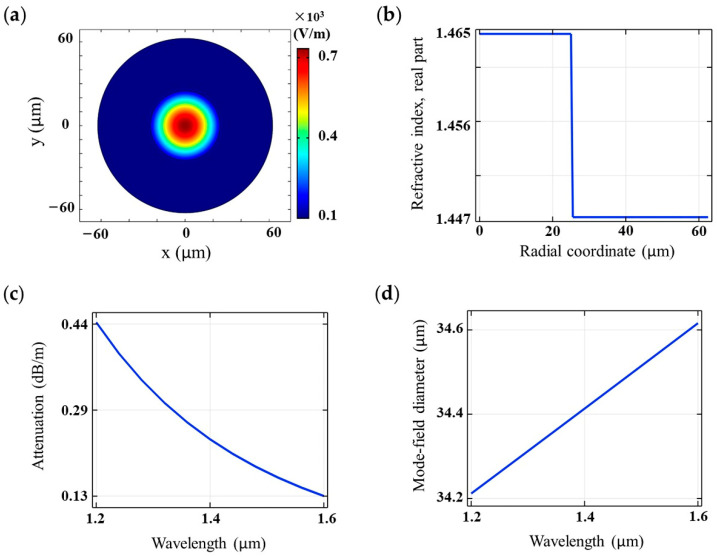
The mode field distribution and characteristic simulation diagrams of the step fiber. (**a**) The mode field distribution diagram of the step fiber. (**b**) The relationship between the effective refractive index (RI) of the step fiber and the radial coordinate. (**c**) The relationship between the confinement loss of the step fiber and the wavelength. (**d**) The relationship between the effective mode field area of the step fiber and the wavelength.

In this paper, the physical parameters of the HC-PCF electromagnetic field were calculated using the wave optical module in the COMSOL Multiphysics software package (COMSOL Inc., Stockholm, Sweden). The fundamental mode of HC-PCF has an electromagnetic field mode similar to the standard step fiber. Figure 2 shows the mode field distribution and characteristic simulation diagrams of the step fiber. The parameters of the step fiber manufactured by Thorlabs (Newtown, NJ, USA) were defined as follows: the core diameter was 50 μm and the cladding diameter was 125 μm; the inner core was made of pure silica glass with *n*_co_ to 1.4457, and the outer layer was coated with cladding material with *n*_cl_ to 1.4378. The working wavelength of this step fiber was 1200–1600 nm.

HC-PCF and the step fiber have similar mode characteristics. When the cut-off frequency *V* < 2.405, HC-PCF transmits the HE_11_ mode, also known as the fundamental mode. When the cut-off frequency *V* > 2.405, the HC-PCF core will appear as high-order modes (HOMs) such as TE_01_ mode, TM_01_ mode, and HE_21_ mode. Since the cut-off frequency is *V* ≠ 0, the fundamental mode HE_11_ of HC-PCFs is the only mode that cannot be cut off. In the finite element characteristic analysis of HC-PCFs, only the HE_11_ mode is generally considered [41]. For the degenerate forms of the HC-PCF fundamental mode, HE*_x_*^11^ and HE*_y_*^11^, although the mode field distribution has a certain asymmetry in different azimuths, the main shape can still be approximated by Gaussian intensity distribution.

### 3.3. Finite Element Simulation of Different Types of HC-PCF

In this paper, COMSOL software was used to simulate and analyze the characteristics of the two kinds of HC-PCF produced by NKT Photonics (Rudersdal, Copenhagen, Denmark) with working wavelengths near the communication C-band, where the working wavelength range of C-band was 1525–1565 nm. HC19-1550 was made by extracting 19 central capillaries and drawing by the tube bundle stacking method. The parameters were as follows: the central hole diameter was 18.5 μm, the spacing of adjacent air holes was 3.8 μm, the diameter of the cladding air hole was 3.5 μm, the optical fiber sensing area diameter was 73.8 μm, and the PML thickness was 5 *λ*. HC-1550-02 was made by extracting 7 central capillaries and drawing by the tube bundle stacking method. The parameters for HC-1550-02 were as follows: the central hole diameter was 9.5 μm, the spacing of adjacent air holes was 3.8 μm, the diameter of the cladding air hole was 3.5 μm, the optical fiber sensing area diameter was 70.8 μm, and the PML thickness was 6 *λ*. In addition, we set the RI of the cladding or PML *n*_PML_ to 1.45, the RI of air hole *n_r_* to 1, and the typical wavelength of PML to *λ*/*n*_PML_. Due to the PBG effect of HC-PCF, *n_eff_* was slightly smaller than *n_r_*, so we selected 0.995 as the mode search reference value. Through analysis and comparison, we eliminated the false solutions of the HC-PCF simulation, and obtained mode field distribution and optical intensity contour distribution diagrams.

**Figure 3:**
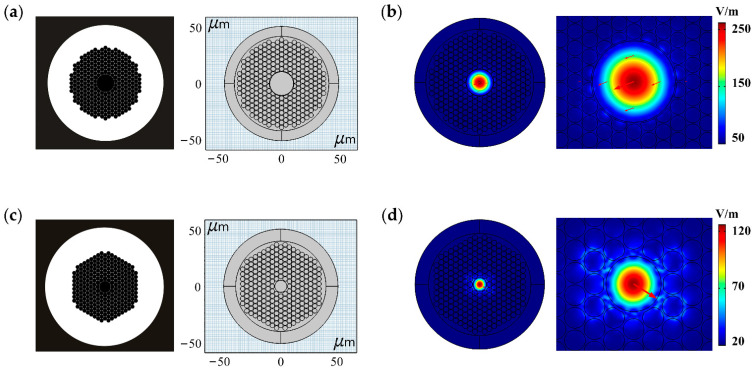
Finite element modeling and simulation results of HC19-1550 and HC-1550-02. (**a**) The cross-sectional and COMSOL simulation modeling schematic diagrams of HC19-1550. (**b**) The mode field diagram of HC19-1550 end-face and the enlarged diagram of HC19-1550 core area. (**c**) The cross-sectional and COMSOL simulation modeling schematic diagrams of HC-1550-02. (**d**) The mode field diagram of HC-1550-02 end-face and the enlarged diagram of HC-1550-02 core area.

Figure 3a,c show the cross-sectional and COMSOL simulation modeling schematic diagrams of HC19-1550 and HC-1550-02. Figure 3b,d present the mode field diagrams of the HC19-1550 and HC-1550-02 end-faces and the enlarged diagrams of these HC-PCF core areas. The red arrows indicate the direction of the electric field on the surface of the fundamental mode.

According to Equation (3), the mode field areas *A_eff_* of the fundamental modes of HC19-1550 and HC-1550-02 are 123.05 μm^2^ and 36.06 μm^2^, respectively. According to Equation (4), the core optical powers *f* of HC19-1550 and HC-1550-02 are 99.51% and 92.83%, respectively. It can be seen that most of the photon energy is restricted to the air hole area of HC-PCF, and it is mainly concentrated in the central air hole, while only a small part of the photon energy is coupled to the quartz substrate material in the cladding. Therefore, it is easier to achieve high-sensitivity sensing with PBG-PCFs than with TIR-PCFs.

**Figure 4:**
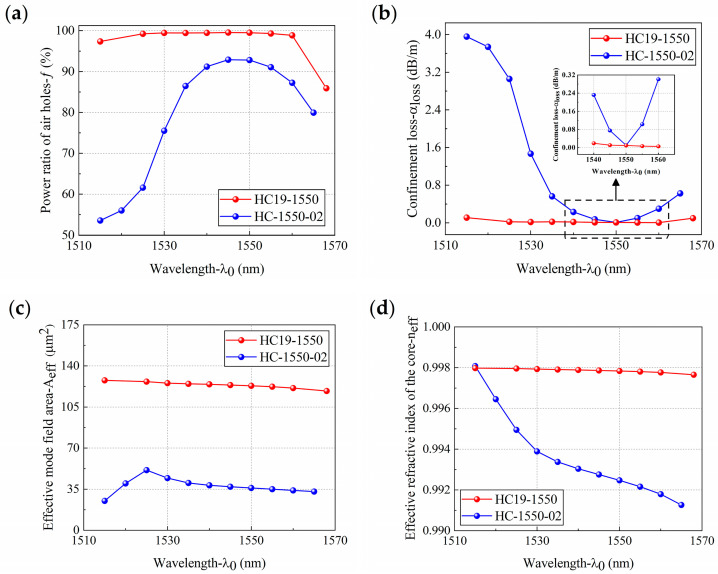
The behavior of (**a**) core optical power, (**b**) confinement loss, (**c**) effective mode field area, and (**d**) effective RI of HC19-1550 and HC-1550-02 in response to changes in wavelength.

To intuitively reflect the behavior of core optical power, confinement loss, effective mode field area, and the effective RI of HC19-1550 and HC-1550-02 in response to changes in wavelength, numerical calculations were carried out in the communication C-band, which is commonly used in sensing (Figure 4).

Figure 4a shows the variation in the core optical power *f* of HC19-1550 and HC-1550-02 in the C-band. It can be seen from Figure 4a that *f* for both first increased and then decreased. On the whole, HC19-1550 had a higher *f*. Within the range of 1515–1560 nm, *f* was above 95% and changed slowly with wavelength. When the wavelength reached 1560 nm, *f* rapidly decreased with wavelength. The *f* of HC-1550-02 was above 85% within the range of 1535–1560 nm. Figure 4b shows the variation of the confinement loss *α*_loss_ of HC19-1550 and HC-1550-02 in the C-band. HC19-1550 had a lower *α*_loss_ overall and changed slowly within the range of 1520–1560 nm. The *α*_loss_ of HC-1550-02 first decreased and then increased with wavelength. Within the range of 1540–1560 nm, *α*_loss_ was below 0.3 dB/m, and the lowest *α*_loss_ was 0.01 dB/m at 1550 nm. In conclusion, HC19-1550 has a wider low confinement loss area than HC-1550-02.

Figure 4c shows the variation in the effective mode field area *A_eff_* of HC19-1550 and HC-1550-02 in the C-band. HC19-1550 had a higher *A_eff_* overall, and it changed slowly with wavelength. The *A_eff_* of HC-1550-02 first increased and then decreased with wavelength, and *A_eff_* reached its maximum value of 51.24 μm^2^ at 1525 nm. When the wavelength reached 1525 nm, *A_eff_* decreased with wavelength. Figure 4d shows the variation in the effective RI *n_eff_* of HC19-1550 and HC-1550-02 in the C-band. The *n_eff_* variation tendency of both decreased with wavelength. On the whole, the *n_eff_* of HC19-1550 was higher and its decrease was slower.

Combined with the variations of the two kinds of HC-PCF characteristics with the wavelength in the C-band, it can be seen that HC19-1550 has a higher core optical power *f*, a lower confinement loss *α*_loss_, a higher effective mode field area *A_eff_*, and a higher effective RI *n_eff_* than HC-1550-02. In addition, its performance is stable. The low confinement loss transmission bandwidths of the two kinds of HC-PCFs were 40 nm and 20 nm, and both had a high *f* and a flat and stable *A_eff_*. In other words, the microstructure of the cladding had the strongest binding ability.

**Figure 5:**
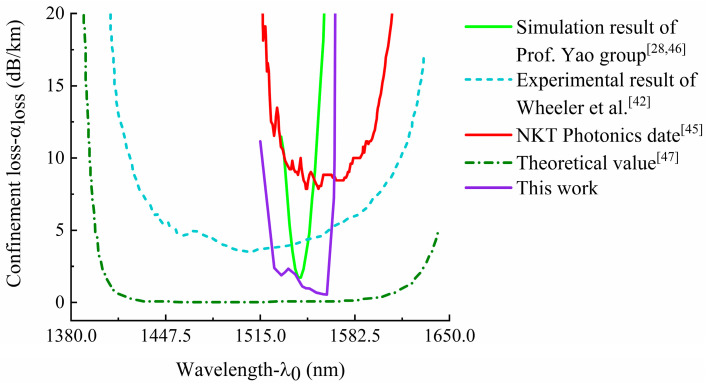
Comparative analysis of simulation result and experimental results for the confinement loss characteristics of HC19-1550.

In order to verify the validity of the simulation model of HC-PCF, we have compared and analyzed the experimental results for the confinement loss characteristics of HC19-1550 from the relevant articles. The experimental results of the relevant articles show that the minimum confinement loss of HC19-1550 can reach 3.5 dB/km near the central wavelength of 1550 nm [42], and the minimum confinement loss of HC-1550-02 can reach 9.5 dB/km [43,44], which is consistent with the simulation results. As shown in Figure 5, we referred to the product manual given by NKT Photonics [45], and referred to the theoretical calculation value and experimental results of the relevant articles. It can be seen that the simulation result of the confinement loss in this paper has a similar trend with the experimental ones, and the order of magnitude of the confinement loss value is the same [46,47], which verifies the validity of the simulation model of HC-PCF in this research.

## 4. Structural Optimization Design of HC-PCF

Through a lot of comparison and analysis, it is found that the main factors affecting the performance of HC-PCF include duty cycle, background material, number of cladding layers, core radius and core quartz-ring thickness [4,17,48]. The air hole of the HC-PCF mentioned above is circular. With the research on HC-PCFs, we have found that optimizing the shape of the core air hole can increase the PBG capability of the PCF. Therefore, the shape of the central air hole designed in this paper is optimized into a rounded hexagonal quartz-ring cladding structure.

### 4.1. Modeling of HC-PCF with Band Gap Cladding Structure

In view of the low cost and wide application of HC-1550-02, here we aimed to optimize the design of the seven-cell HC-1550-02. This HC-PCF was made up of eight-layer air holes that were arranged in a triangular lattice, and a hexagonal air hole cladding structure was formed after rounding. The central air hole was made by removing seven quartz capillaries and drawing by the tube bundle stacking method [49,50]. The variable parameters of HC-PCF were defined as follows: the spacing of adjacent air holes was *Λ*, the diameter of the cladding air hole was *d*, the core radius was *R*, the actual core quartz-ring thickness was *t*, and the relative thickness of the core quartz-ring was *T*. Generally, we use the relative thickness of the core quartz-ring to describe the PBG capability of the HC-PCF. The expression of *T* is as follows:

(6) T=t/(Λ−d)

**Figure 6:**
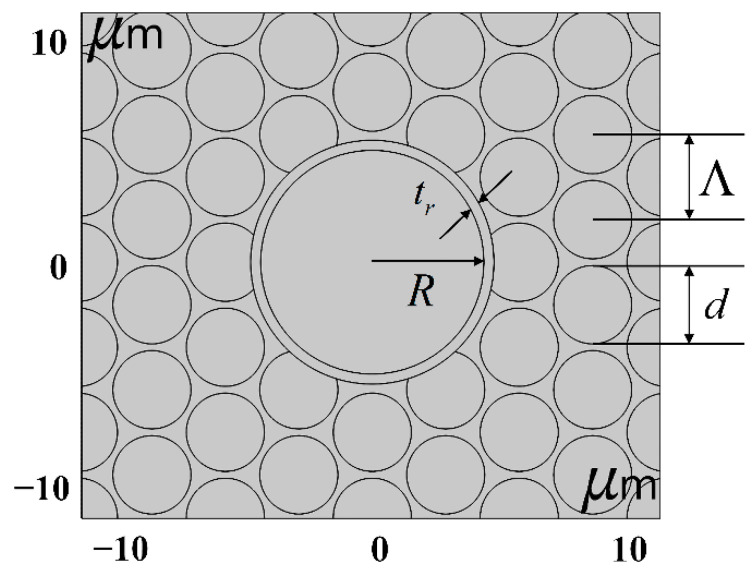
The basic structural schematic diagram of hollow-core photonic crystal fiber (HC-PCF) with a band gap cladding structure.

In this paper, the effects of the core radius and the core quartz-ring thickness on the performance of HC-PCFs were analyzed by selecting the wavelength *λ* = 1550 nm as a reference, where *λ* = 1550 nm is regarded as a spectral window for optical fibers to achieve ultra-long-distance transmission. The basic structural schematic diagram of HC-PCF with band gap cladding structure is shown in Figure 6. The variable parameters were as follows: *Λ* was 3.8 μm, *d* was 3.5 μm, *R* was 5 μm, *t* was 0.45 μm, and *T* was 1.5. Since *R* refers to the radius from the core to the inner quartz-ring wall, changing the core quartz-ring thickness will not affect the value of *R*.

### 4.2. Effects of the Core Radius on HC-PCF Performance

**Figure 7:**
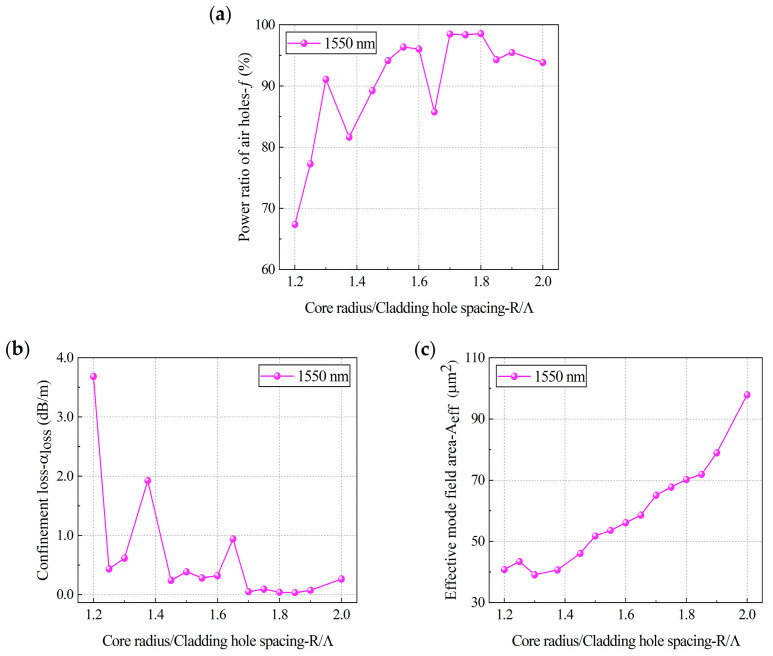
The response of (**a**) core optical power, (**b**) confinement loss and (**c**) effective mode field area of HC-PCF with a band gap cladding structure to changes in the core radius.

Under the condition that other parameters remain unchanged, only the size of the core radius *R* was changed to obtain the effects of *R*/*Λ* on HC-PCF performance, as shown in Figure 7.

Figure 7a shows the relationship between the core optical power *f* and *R*/*Λ*. It was found that when *R*/*Λ* = 1.2, *f* was only 67.41%, while at *R*/*Λ* = 1.3, *f* rose rapidly to 91.13%. When *R*/*Λ* changed from 1.3 to 1.7, there were two large fluctuations in *f*. When *R*/*Λ* changed from 1.7 to 1.8, *f* did not fluctuate, and *f* reached the maximum value of 98.58% at *R*/*Λ* = 1.8. When *R*/*Λ* changed from 1.8 to 2.0, *f* slightly decreased.

**Figure 8:**
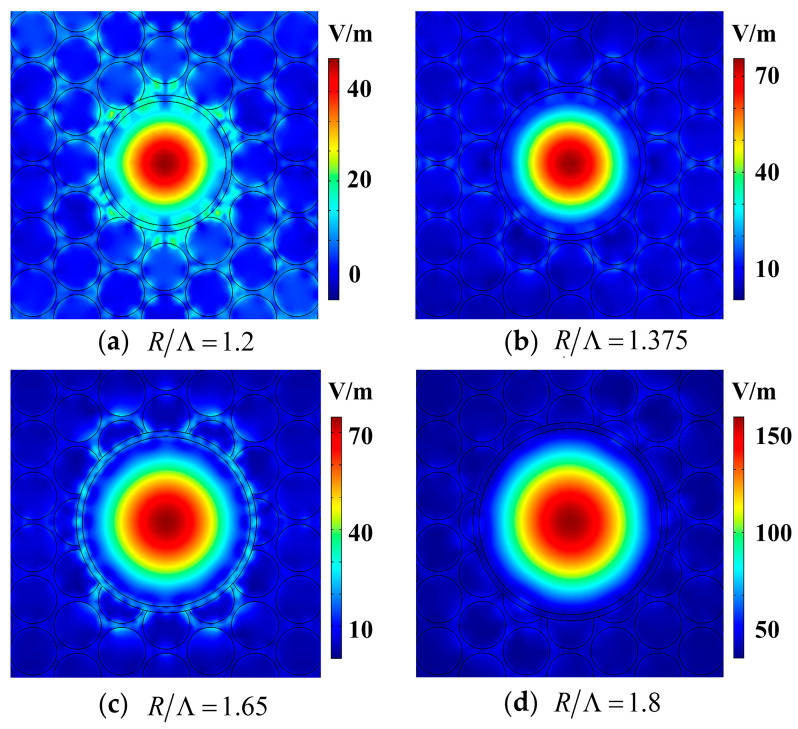
The mode field distribution diagrams of HC-PCF when (**a**) *R*/*Λ* = 1.2, (**b**) *R*/*Λ* = 1.375, (**c**) *R*/*Λ* = 1.65, and (**d**) *R*/*Λ* = 1.8.

To further analyze the cause of fluctuations, the mode field distribution diagrams when *R*/*Λ* = 1.2, 1.375, 1.65, and 1.8 were selected, as shown in Figure 8. According to the mode field distribution diagrams of HC-PCFs, when *R*/*Λ* was 1.2 and 1.375, *f* was 67.41% and 81.69%, respectively. It can be seen that the transmitted light is located at the edge of the PBG, which leads to the light being diffused into the cladding that cannot be reflected back to the core, with part of the photon energy being transmitted into the cladding, forming a relatively obvious surface mode. When *R*/*Λ* was 1.65, *f* was 85.78%; it can be seen that the amount of photon energy diffused into the cladding significantly reduced, but a significant amount of photon energy accumulated in the quartz-ring. The fundamental mode and the surface mode are coupled near the core quartz-ring, resulting in a significant reduction in the surface mode area being diffused into the cladding. When *R*/*Λ* was 1.8, *f* was 98.58%, and it can be seen that the surface mode of the cladding disappears; that is, the fundamental mode and the surface mode are not coupled in the core quartz-ring, and almost all of the energy was concentrated in the core. *R*/*Λ* had the best effect on *f* under this condition.

From the relationship between the confinement loss *α*_loss_ and *R*/*Λ* (Figure 7b), it can be seen that, except for the fluctuations of some points, *α*_loss_ decreased rapidly with increased *R*/*Λ*, *α*_loss_ fluctuated twice, and the final *α*_loss_ reached a stable level. When *R*/*Λ* was 1.2, *α*_loss_ was 3.69 dB/m; with this structure, the HC-PCF had no practical value. With the increase of *R*/*Λ*, *α*_loss_ decreased rapidly. When *R*/*Λ* was at 1.375 and 1.65, there were large fluctuations. There may be two reasons for this phenomenon [51]: one is that the coupling between the fundamental mode and the surface mode causes the core energy to decrease; the other is the intrinsic absorption of the material. Since *R*/*Λ* in Figure 7a,b exhibited large fluctuations at 1.375 and 1.65, respectively, it is certain that the intrinsic absorption of the material had an impact. As for the coupling between the fundamental mode and the surface mode, this needs to be further determined through the relationship between the effective mode field area *A_eff_* and *R*/*Λ*. When *R*/*Λ* changed from 1.7 to 1.9, *α*_loss_ changed slowly with the core radius, and *α*_loss_ reached the lowest value of 0.038 dB/m at *R*/*Λ* = 1.85. When *R*/*Λ* changed from 1.85 to 2.0, *α*_loss_ increased slightly.

Figure 7c shows the relationship between the effective mode field area *A_eff_* and *R*/*Λ*. It can be seen that, except for a fluctuation phenomenon at *R*/*Λ* = 1.25, the overall variation trend was a gradual increase with increased *R*/*Λ*, and the upward trend significantly accelerated after *R*/*Λ* = 1.85. As also demonstrated by Figure 7a, the reason for this acceleration is that the photon energy is located at the edge of the band gap.

Similarly, we also analyzed the reasons for the fluctuation when *R*/*Λ* was 1.25, 1.375 and 1.65 by Figure 7. When *R*/*Λ* was 1.25, only the variation trend of *A_eff_* fluctuated, so it can be inferred that the reason for the fluctuation is the increase of *A_eff_* because of the coupling between the fundamental mode and the surface mode. At *R*/*Λ* = 1.375 and *R*/*Λ* = 1.65, the variation trend of *A_eff_* did not change suddenly, so it can be inferred that the fluctuations of *f* and *α*_loss_ were solely caused by the intrinsic absorption of the material.

In conclusion, the core radius *R* had a relatively large impact on the core optical power *f*, the confinement loss *α*_loss_, and the effective mode field area *A_eff_*. When *R*/*Λ* changed from 1.5 to 1.6 and from 1.7 to 1.9, *f* was higher and *α*_loss_ was lower. Therefore, *R*/*Λ* = 1.8 was selected as the optimal parameter to study the effects of the relative thickness of the core quartz-ring on HC-PCF performance.

### 4.3. Effects of the Relative Thickness of the Core Quartz-Ring on HC-PCF Performance

**Figure 9:**
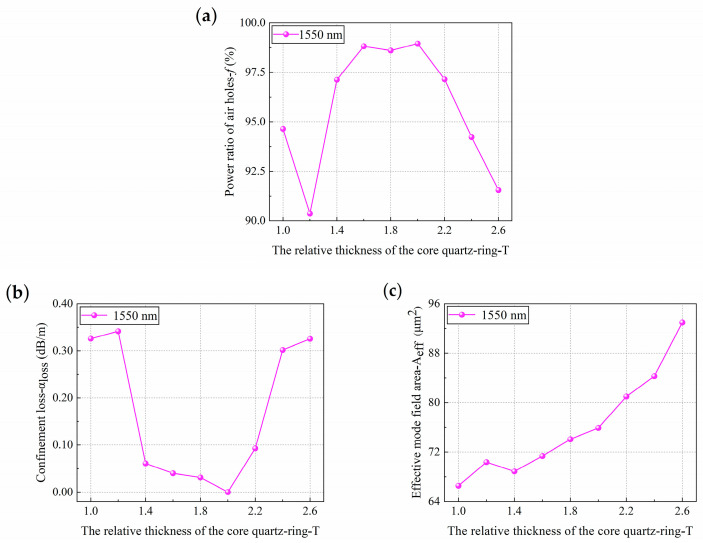
The response of (**a**) core optical power, (**b**) confinement loss and (**c**) effective mode field area of HC-PCF with band gap cladding structure to change in the relative thickness of the core quartz-ring.

Under the condition that other parameters remain unchanged, only the actual core quartz-ring thickness *t* was changed to obtain the effects of *T* on HC-PCF performance, as shown in Figure 9.

From the relationship between the core optical power *f* and the relative thickness of the core quartz-ring *T*, as shown in Figure 9a, it can be seen that, except for a fluctuation phenomenon at *T* = 1.2, *f* first increased and then decreased with the increase of *T*. When *T* changed from 1.4 to 2.2, *f* was above 95%. While *T* changed from 1.6 to 2.0, *f* changed slowly with the increase of *T*, and *f* reached the maximum value of 98.95% at *T* = 2.0. When *T* began at 2.0, *f* decreased rapidly with the increase of *T*.

From the relationship between the confinement loss *α*_loss_ and the relative thickness of the core quartz-ring *T*, as shown in Figure 9b, it can be seen that the overall variation trend of *α*_loss_ was that it first decreased and then increased with the increase of *T*. When *T* changed from 1.4 to 2.2, *α*_loss_ was below 0.10 dB/m, and *α*_loss_ reached the lowest value of 7.31 × 10^−5^ dB/m at *T* = 2.0. Combined with the results from Figure 9a, it can be concluded that the lowest transmission loss band in the optical fiber had a higher *f*.

From the relationship between the effective mode field area *A_eff_* and the relative thickness of the core quartz-ring *T*, as shown in Figure 9c, it can be seen that *A_eff_* increased with the increase of *T*, and its trend increased rapidly when *T* > 2.0. As also shown in Figure 9a,b, *f* decreased rapidly and *α*_loss_ increased rapidly when *T* > 2.0. It can be inferred that the reason for these changes was the coupling between the fundamental mode and the surface mode, which caused most of the photon energy to be diffused into the cladding for transmission, so the higher *T* had a relatively large impact on the bandwidth.

As shown in Figure 9, a fluctuation phenomenon occurred at *T* = 1.2. Since *A_eff_* also has a slight fluctuation at *T* = 1.2 in Figure 9c, it can be inferred that this fluctuation phenomenon was caused by the interaction of the intrinsic absorption and the coupling between the fundamental mode and the surface mode. In conclusion, when *T* changed from 1.6 to 2.0, *f* was higher and *α*_loss_ was lower, and the results of *f* and *α*_loss_ are optimal when *T* = 2.0. Therefore, *T* = 2.0 was selected as the final optimized structure in this paper.

## 5. Numerical Results and Discussion

### 5.1. Characteristic Analysis of the Optimized HC-PCF

**Figure 10:**
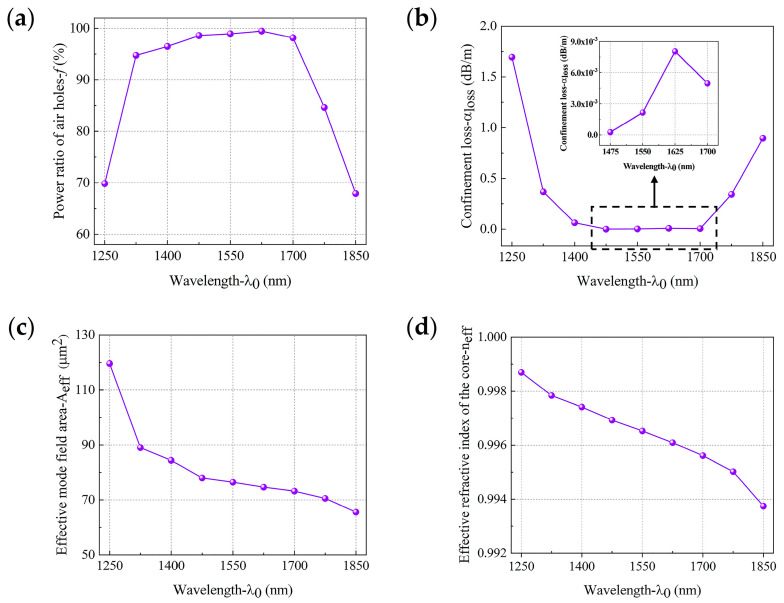
The response of (**a**) core optical power, (**b**) confinement loss, (**c**) effective mode field area, and (**d**) effective RI of the optimized HC-PCF to changes in wavelength.

**Figure 11:**
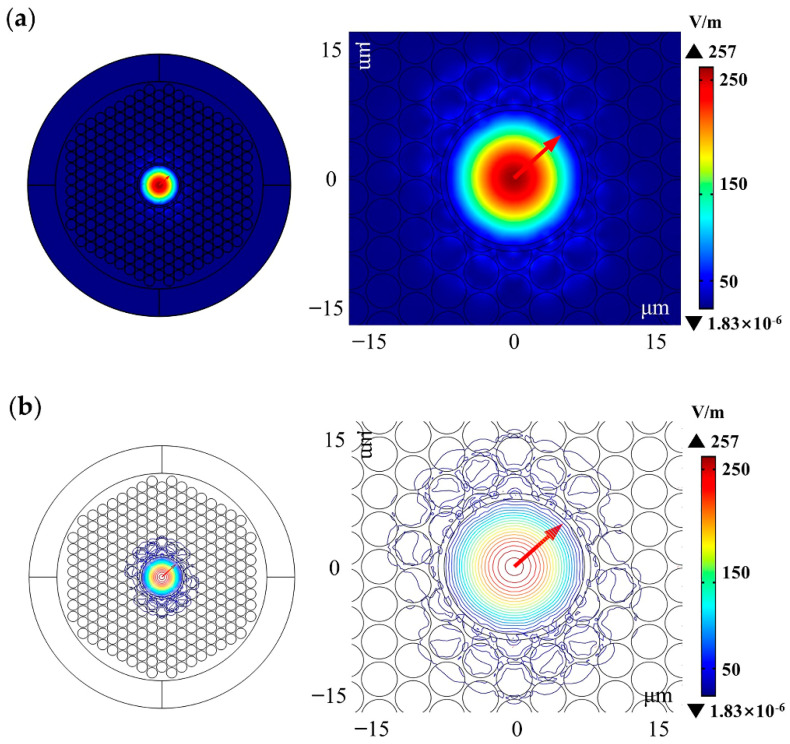
The optimized HC-PCF (**a**) mode field distribution diagram and (**b**) optical intensity contour distribution diagram at a wavelength of 1550 nm.

Figure 10 presents the relationship between the optimized HC-PCF characteristics and the wavelength. It can be seen that, *f* is above 98% and *α*_loss_ is below 9.0 × 10^−3^ dB/m within the range of 1475–1700 nm, and the variation range of *A_eff_* does not exceed 10 μm^2^ within the range of 1475–1775 nm. It can be seen from Figure 10d that *n_eff_* decreased with the wavelength under this structure, indicating that the low confinement loss bandwidth of this structure is at least in the range of 225 nm, while the transmission bandwidth of the traditional HC-PCFs is only in the order of tens of nanometers. The optimized HC-PCF mode field distribution diagrams and optical intensity contour distribution diagrams at 1550 nm are shown in Figure 11. It was found that almost all of the energy was concentrated in the core.

### 5.2. The Relative Sensitivity of the Designed HC-PCF Sensor

The relative sensitivity of gas or liquid sensors using HC-PCF as the transmission medium can be calculated by the intensity of the light-matter interaction, which depends on the absorption coefficient at a particular frequency. According to the Beer–Lambert law, it can be defined as follows [52,53]:

(7) $$I_{T}\left(\omega \right)=I_{0}\left(\omega \right)\exp\left[-\gamma \alpha \left(\omega \right)l_{c}\right]$$

where *I_T_* (*ω*) and *I_0_* (*ω*) are the output light intensities with and without the presence of the analyte needed to be detected, *ω* is the operating frequency of monochromatic light, *γ* is the relative sensitivity, *α*(*ω*) indicates the absorption coefficient of the measured analyte, *l_c_* is the effective absorption length of the gas or liquid channel.

The absorbance of the analyte to be detected can be calculated by [54]:

(8) $$A=\log\left(\frac{I_{T}\left(\omega \right)}{I_{0}\left(\omega \right)}\right)=-\gamma \alpha \left(\omega \right)l_{c}$$

Now, to investigate the sensing performance of the designed HC-PCF, the relative sensitivity is considered as a fundamental sensing representative which is calculated as follows [55]:

(9) $$\gamma =\left(n_{r}/n_{eff}\right)\cdot f$$

where *n_r_* represents the RI of the analyte needed to be detected (which is 1 in the case of air), and *f* is the optical power distribution function, referring to Equation (4).

**Figure 12:**
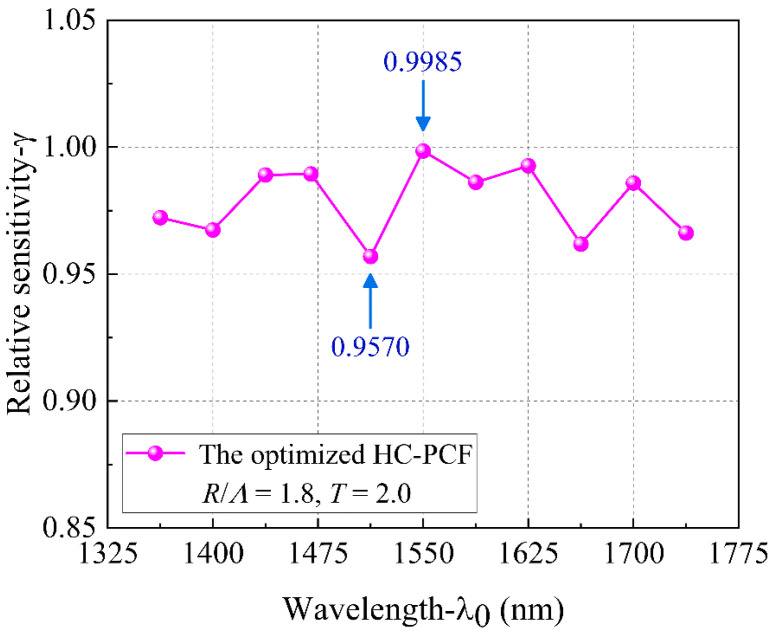
The relative sensitivity of the optimized HC-PCF in the range of the low confinement loss bandwidth.

As can be seen from the simulation results in Figure 10 that the *f* value of HC-PCFs with band gap cladding structure has been greatly increased. The higher the *f* value, the higher the *γ* value, and the higher the sensitivity of the designed sensor. Figure 12 shows that the relative sensitivity of the optimized HC-PCF in the range of the low confinement loss bandwidth. Numerical results show that the relative sensitivity of the optimized HC-PCF in the range of the low confinement loss bandwidth is above 0.9570, and the highest relative sensitivity is 0.9985 can be obtained at 1550 nm. Compared with the ordinary circular central air hole structure with *γ* = 0.9435 at 1550 nm, the relative sensitivity of the optimized HC-PCF is further improved and is closer to the theoretical limit.

Compared with other similar works, the designed sensor exhibits superior relative sensitivity and lower confinement loss, as shown in Table 1.

## 6. Conclusions

HC-PCFs with a band gap cladding structure can be used in many fields, including active optical devices, communications, optical fiber sensing, and other industrial clusters, due to its flexible structure and excellent optical characteristics. Based on the special optical transmission characteristics of HC-PCFs and the photonic band gap cladding structure, finite element simulation and structural optimization design were carried out to reveal the optical transmission characteristics of HC-PCFs. We measured the core optical power, confinement loss, and effective mode field area. The optimal optical performance parameters were determined by changing the core radius and the core quartz-ring thickness of the HC-PCF band gap cladding structure.

The following conclusions can be drawn from this research:

(1) HC19-1550 has a higher core optical power, lower confinement loss, higher effective mode field area, and higher effective refractive index than HC-1550-02, and its performance is stable. The low confinement loss transmission bandwidths of HC19-1550 and HC-1550-02 are 40 nm and 20 nm, respectively, and both have a high core optical power and a flat and stable effective mode field area.
(2) The core radius and the relative thickness of the core quartz-ring have a relatively large impact on the core optical power, confinement loss, and effective mode field area. When the core radius changed from 1.5 to 1.6 times the spacing of adjacent air holes, and from 1.7 to 1.9 times the spacing of adjacent air holes, the core optical power was higher and the confinement loss was lower. When the relative thickness of the core quartz-ring changed from 1.6 to 2.0 times the spacing of adjacent air holes, the core optical power was higher and the confinement loss was lower, and when the relative thickness of the core quartz-ring was 2.0, the optimal core optical power and confinement loss results were obtained.
(3) The optimal number of cladding layers is eight, the optimal core radius is 1.8 times the spacing of adjacent air holes, and the optimal-relative thickness of the core quartz-ring is 2.0. This optimized structure achieves an ultra-high core optical power and almost zero confinement loss.
(4) The low confinement loss bandwidth of the optimized structure is 225 nm, while the transmission bandwidth of the traditional HC-PCF is in the range of tens of nanometers. In addition, in terms of the transmission bandwidth of the optimized structure, the core optical power is above 98%, the confinement loss is below 9.0 × 10^−3^ dB/m, the variation range of the effective mode field area does not exceed 10 μm^2^, and the relative sensitivity is above 0.9570. Compared with the ordinary circular central air hole structure, the relative sensitivity of the optimized HC-PCF is further improved and is closer to the theoretical limit. Therefore, the sensor with this design structure is highly suitable for high-sensitivity gas or liquid sensing.

## Figures and Tables

**Table 1:** The assessment table among our reported HC-PCF structure and the previous published prior PCFs of their optical properties such as the relative sensitivity, the confinement loss at their operating region and also shows their design of fiber.

Prior in PCFs	Operating Region	Relative Sensitivity (%)/Analyte	Confinement Loss (dB/m)	Design of Structure	
				Cladding	Core
Ref. [56]	*λ* = 1.56 μm	99.98/Air	2.811 × 10^−4^	Floriated hole	Circular
Ref. [57]	*λ* = 1.50 μm	63.40/Sulfuric acid	1.422 × 10^−^^17^	Elliptical holes	Hexagonal
Ref. [58]	*λ* = 1.55 μm	58.3/Propane	9.2 × 10^−^^7^	Spiral holes	Elliptical
		62.7/Propylene	7.1 × 10^−^^7^		
Ref. [59]	*λ* = 1.25 μm	96.198/Sorbitol	2.24 × 10^−^^9^	Circular holes	Hexagonal
		94.124/Sorbitol	1.67 × 10^−^^10^	Hexagonal holes	
Ref. [60]	*λ* = 1.33 μm	53.07/N_2_ & N_2_H_2_	1.26 × 10^−^^5^	Circular holes	Rectangular slot
Ref. [61]	*λ* = 1.33 μm	88.75/Saccharin	1.8414 × 10^−^^15^	Hexagonal holes	Hexagonal
		87.37/Sorbitol	1.5462 × 10^−^^14^		
		86.72/Butyl Acetate	6.3245 × 10^−^^13^		
Ref. [62]	*f_0_*^1^ = 1 THz	81.46/Ethanol	5.85 × 10^−^^8^	Hexagonal holes	Hexagonal
		82.26/Benzene	6.07 × 10^−^^8^		
		79.22/Water	5.84 × 10^−^^8^		
Ref. [63]	*f_0_* = 2 THz	93.50/Red blood cells	3.11 × 10^−^^14^	Rectangular holes	Rectangular
This work	*λ* = 1.55 μm	99.85/Air	7.31 × 10^−5^	Circular holes	Quartz-Ring

^1^*f_0_* represents frequency.

## Data Availability

Data sharing not applicable.
